# Trends and factors associated with recent HIV testing among women in Haiti: a cross-sectional study using data from nationally representative surveys

**DOI:** 10.1186/s12879-023-08936-z

**Published:** 2024-01-11

**Authors:** Fanor Joseph, David Jean Simon, Vénunyé Claude Kondo Tokpovi, Ann Kiragu, Marie-Reine Ayawavi Sitsope Toudeka, Roodjmie Nazaire

**Affiliations:** 1 Doctoral School of Social and Human Sciences, University of Antananarivo, Antananarivo, Madagascar; 2 Bureau d’Etudes Et de Recherche en Statistiques Appliquées, Suivi Et Evaluation (BERSA-SE), Port-au-Prince, Haiti; 3 Groupe de Recherche Sur L’inadaptation Psychosociale (GRIP), University of Laval, Laval, Canada; 4 Department of Law and Political and Social Sciences, University of Sorbonne Paris Nord, Paris, France; 5 Unité de Recherche Démographique de l’Université de Lomé, Lome, Togo; 6 Faculté de Médecine et de Pharmacie (FMP), Université d’Etat d’Haïti (UEH), Port-Au-Prince, Haiti

**Keywords:** HIV testing, Haiti, HDHS, Women, Trends, Factors

## Abstract

**Introduction:**

In the Latin America and Caribbean region, Haiti is one of the countries with the highest rates of HIV. Therefore, this study examined the factors associated with HIV testing among women in Haiti and trends in HIV testing in 2006, 2012, and 2016/17.

**Methods:**

Data from the last three Haitian Demographic and Health Surveys (2006, 2012, and 2016/17) were used. The analysis was restricted to women aged of 15–49 years who made their sexual debut. STATA/SE 16.0 was employed to analyze the data by computing descriptive statistics, Chi‑square, and multilevel regression model to describe the trends and identify factors associated with HIV testing in Haiti. *P*-value less than 0.05 was taken as a significant association.

**Results:**

HIV testing prevalence increased more than twofold from 2006 (8.8%) to 2017 (21.3%); however, it decreased by 11.6% between 2012 and 2016/17. Additionally, the results indicated that age, place of residence, region, education level, wealth index, mass media exposure, marital status, health insurance, age at first sex and number of sexual partners were significantly associated with HIV testing.

**Conclusions:**

To significantly increase HIV testing prevalence among women, the Haitian government must invest much more in their health education while targeting vulnerable groups (youth, women in union, and women with low economic status).

**Supplementary Information:**

The online version contains supplementary material available at 10.1186/s12879-023-08936-z.

## Introduction

HIV/AIDS remain a major public health issue and preventing HIV infection, a global public health goal. According to the United Nations Joint Programme on HIV/AIDS (UNAIDS), 38.4 million people were living with HIV (PLWH) [1]. Also, it is reported that more than two-thirds of PLWH were from Eastern and southern Africa [1]. This region was responsible for 670,000 of the 1.5 million new infections and 280,000 of the 650,000 AIDS-related deaths reported globally in 2021 [2].

The Caribbean is the second-most affected region in the world in terms of HIV. Based on recent data, the estimated HIV prevalence among adults was 1.2% in this region, with 14,000 people newly infected in 2021 [1]. Haiti alone accounts for nearly half of new HIV infections and deaths due to AIDS-related illness [3]. Despite significant efforts in reducing new HIV infections over the past two decades, the number of PLWH remains high in Haiti. Over 150,000 people live with HIV, representing a prevalence of 1.8% [4]. Among PLWH, a third do not know their serostatus, and 58% are not receiving antiretroviral treatment [2]. Moreover, it should be noted that HIV infection is disproportionally distributed by gender in Haiti. HIV prevalence is 2.2% in female, compared with 1.4% in male [1].

Routine testing for HIV is one of the key strategies in HIV/AIDS prevention and control [5]; it is also an essential step in detecting the virus and accessing treatment services to receive life-saving antiretroviral therapy in case of a positive diagnosis [5, 6]. However, in some regions, particularly in Latin America and the Caribbean, efforts to ensure timely diagnosis and enrollment in care remain insufficient [7]. Nonetheless, in Haiti, to strengthen HIV/AIDS response and intensify efforts in prevention and care, a key element of HIV surveillance is the National HIV Reporting Electronic Platform, a longitudinal HIV case-based surveillance (CBS) system known as "Suivi Actif Longitudinal du VIH en Haïti (SALVH)" [8] which captures and systematically reports newly diagnosed HIV cases and sentinel events, integrating data from multiple sources into a single national dataset [9].

HIV testing is an important step for diagnosis and eventually access to care and treatment, and various factors are associated to its uptake. Studies have found that socioeconomic factors such as educational attainment and income influence HIV testing [10–12]. The level of education is linked to the type of employment leading to better living conditions and access to healthcare [13–15] particularly in urbans areas [16, 17]. Similarly, HIV related awareness has been linked to testing [18]. Moreover, studies in sub-Saharan Africa have found demographic factors particularly age [19, 20] and age at first sex were significantly associated with HIV testing [21, 22]. HIV testing has also been found to be more common among people who engage in behaviors that increase HIV risk such as multiple sexual partners [11, 23–25].

Early diagnosis is crucial for successful treatment of HIV. Failure to uptake a HIV test can lead to chronic HIV infection exposing the individual to progressive exhaustion of the immune system [26]. Evidence suggests that an early diagnosis through testing can halt the immune disfunction with the initiation of antiretroviral therapy [27]. Additionally, studies have shown that severe infection increases cardiovascular risks among HIV-infected individuals [28].

Studies on HIV testing are very limited in Haiti (focused only on men) [29]; however, they are important for informing strategies and programs to respond to HIV/AIDS. To partially fill this gap, this study aims to examine the trends and factors associated with recent HIV testing among women in Haiti, a population at high risk of HIV infection.

## Materials and methods

### Study setting

Located on the western one-third of the island of Hispaniola in the Caribbean Sea, Haiti has a population of approximately 11,7 million people [30]. Administratively, the republic of Haiti is divided into 10 departments (Ouest, Sud, Sud-Est, Grande Anse, Nippes, Nord, Nord-Ouest, Nord-Est, Centre, and Artibonite), 41 districts, 140 municipalities and 570 communal sections [31]. Haiti has a very young population structure: the median age is estimated at 24 years and less than 5% of people is 65 years or older [30]. Economically, Haiti is the Western Hemisphere's poorest country with a GDP per capita of 1829.6 (current USD) [32]. Further, about 25% live below the national extreme poverty line ($1.23 per day) [33] with a life expectancy at birth of 64.7 years [34]. Haiti is also very vulnerable in terms of access to healthcare. Recent WHO estimates revealed that the country provides an average of 7 hospital beds, 2.3 doctors, and 4 nurses and midwives per 10,000 inhabitants, compared with 15.6, 14.5 and 13.3 respectively in neighbouring Dominican Republic [35–37].

### Data source and sample design

Data for this study were extracted from the last three Haitian Demographic and Health Surveys (HDHS: 2006, 2012, and 2016/17). Note that five DHS have already been conducted in Haiti; however, information on HIV testing was not collected for the first two HDHS (1994/1995 and 2000). The HDHS are nationally representative household surveys conducted by the Haitian Institute for Children with technical support from global partners including the Haitian Bureau of Statistics, the Ministry of Public Health and Population, and the ICF through the DHS Program of the United States Agency for International Development (USAID). The main objective of these surveys was to provide information on women’s fertility, childhood mortality, contraceptive use, sexually transmitted infections, and maternal and child health issues in Haiti [31].

A two-stage stratified sampling design was applied that involved randomly selecting the sampling clusters that were created in the first stage, followed by randomly selecting households per cluster with equal probabilities in a systematic approach in the second stage. Four questionnaires were used for the data collection: Household Questionnaire, Women’s Questionnaire, Men’s Questionnaire and Biomarker Questionnaire. Detailed information regarding the HDHS sampling and data collection have been published elsewhere [31]. For this study, we extracted all relevant variables from the women data files (individual recode) in the 2006, 2012, and 2016/17 HDHS data sets. The data analysed in this present research work relate to women of childbearing age who have already had sexual intercourse.

### Study population

The study population consisted of women aged of 15–49 years who made their sexual debut, as this is one of the groups at a higher HIV risk in Haiti [38]. During the last three HDHS, 10,757, 14,287, and 14,371 women were successfully interviewed respectively. Of the total women who participated, 8627, 11,637, and 11,892 reported that they have had their first sexual intercourse, respectively.

### Study variables

#### Dependent variable

The outcome variable of interest for this study was HIV testing. Generated from the items "Last time tested for HIV" and "Received result from last HIV test", it was coded "yes" if a woman declared testing for HIV last 12 months prior the survey and receiving result, and "no" otherwise.

#### Independent variables

Based on existing literature [10, 25, 39–41] as well as their availability in the Women dataset, we identified potential factors that could influence HIV testing among women in Haiti at two levels (individual and community). The individual level variables included age ("less than 25 years", "25–34", "35 and above"), religion ("Christian", "Non-Christian"), education level ("primary or less", "secondary", and "higher"), frequency of listening to radio ("not at all", "less than once a week", "at least once a week", "almost every day"), frequency of watching TV ("not at all", "less than once a week", "at least once a week", "almost every day"), currently working ("yes", "no"), being covered by health insurance ("yes", "no"), marital status ("never married", "in union", "widowed/divorced/separated"), age at first sex ("less than 15", "15–18", "18 and above"), number of sexual partners ("no partner", "one partner", "two and above"), had any STD last 12 months ("yes", "no"), and wealth index ("poorest", "poorer", "middle", "richer", "richest"). A principal component analysis, where individual households were positioned on a continuous scale of relative wealth, was used to generate the wealth index. Detailed information about the wealth index construction can be found in the HDHS reports [31].

Place of residence and region were considered as community-level variables. Place of residence and region are criteria utilized in designing the sample to estimate the prevalence of core demographic and health indicators at the national level. Place of residence was divided into "rural" and "urban" and region was coded as "Ouest", "Sud-Est", "Nord", "Nord-Est", "Artibonite", "Centre", "Sud", "Grand’Anse/Nippes", and "Nord-Ouest". These two variables directly explain community characteristics.

### Statistical analysis

Data analysis was performed at three levels: descriptive, bivariate and multivariate. Descriptive univariate analyses illustrated frequencies and percentages to describe the women’s profile. Trend analysis of HIV testing among women was conducted relative to the respective survey years (2006, 2012, and 2016/17). Bivariate analyses (cross-tabulations with chi-square tests) were carried out separately for each dataset to examine the association between HIV testing and the selected independent variables using *p*-value < 0.05 as cut of points. In order to assess the effects of several identified individual and community-level factors associated with HIV testing, multilevel analyses (two-level mixed-effects logistic regression model) were applied since the HDHS data were hierarchical (individual "level 1" variables were nested within community "level 2" variables) [42]. For each dataset, we have fitted four models: null model (Model-0), model 1 (Model-I), model 2 (Model-II), and model 3 (Model-III). The null model was fitted with only the outcome variable [42]. Model 1, model 2, and model 3 were fitted using individual-level variables, community-level variables, and both individual and community-level variables respectively. The "melogit" command was used in Stata software to account for the clustering of the outcome variable within and across sampling clusters of the survey design. Results of fixed effects were reported as adjusted odds ratios (aOR) with their corresponding 95% confidence intervals (CI). The random effect was interpreted using the Intra-class Correlation Coefficient (ICC) and the Proportional Change in Variance (PCV) and compared across the progressive models by looking at them. Moreover, the variance inflation factor (VIF) was used to assess multi-collinearity. None of the variables displayed multi-collinearity problems (all VIF < 5) [43–45]. Log-likelihood and Akaike Information Criterion (AIC) were used to verify model fitness, and a model with the highest log-likelihood and lowest AIC has been deemed as a best-fit model [46]. All analyses were weighted to get unbiased estimates, and carried out in STATA 16.0 software (Stata Corp, Tex, USA) using "*svy*" command to adjust for the complex sampling structure of the data. Statistical significance was declared at *p* < 0.05.

#### Demographic decomposition analysis

To examine changes in HIV testing prevalence between 2006 and 2016/17, demographic decomposition analysis was used. Specifically, this method allows us to examine how a change in the dependent variable is driven by changes in each independent variable (reflecting a group or a process). In a demographic decomposition, the main question is about the contribution of "composition" or group-specific size vs "group-specific behavior" [47].

Formally, the national prevalence of HIV testing (Y) is expressed as a weighted average (by ${\omega }_{j}$) of HIV testing prevalence in subpopulations groups defined by the selected independent variables categories (${y}_{j}$).

1 $${Y}_{t}=\sum {\omega }_{jt}*{y}_{jt}$$

In this formula, a national change in the HIV testing prevalence can be broken down into two components:2

$$Y$$: dependent variable.

$\omega$: demographic weight of an individual subgroup.

${y}_{j}$: value of the dependent variable for the group $j$

$$\overline{{y}_{j}}$$: $$\left[{(y}_{j (t+1)}+{y}_{j(t)})/2\right]$$

$$t$$: time.

$\Delta$: indicates the change.

The compositional effect is change that is driven by changes in the relative size of each subgroup, while the second component of change reflects changes in the behaviors of each subgroup [48].

It is important to mention that the formula (2) (basic decomposition) can be extended by noting that the behavioural of a given group (j) can be expressed as a function of one or more other variables [48]. For this, let’s use a simple regression analysis where:

3 $${y}_{j}=\alpha +\beta {X}_{j}+{\mu }_{j}$$

$$X$$: independent variable.

$\alpha$: intercept or baseline value.

$\beta$: the marginal change in $Y$ associated with a one-unit change of $X$

$${\mu }_{j}$$: error term.

In this case, the change of ${y}_{j}$ between two periods $(t, t+1)$ is expressed as follows:

4 $$\Delta {y}_{j}=\Delta \alpha +\overline{\beta }\Delta {x}_{j}+\overline{{x}_{j}}\Delta \beta +\Delta {\mu }_{j}$$

If the categories of $x$ do not change between $t$ and $t+1$, the second term of this equation is null, and $\overline{x}$ is equal to $x$ [48]. The equation thus reduces to:

5 $$\Delta {y}_{j}=\Delta \alpha +{X}_{j}\Delta \beta +\Delta {\mu }_{j}$$

Inserting (5) in (2), we obtain:6

This new decomposition is called advanced decomposition. It allows to identify, in greater detail, the groups driving the change (referred to here as the compositional effect) and also consider how changes in the behavior of each group mattered (referred to here as the behavioral effect) [47, 48].

In our demographic decomposition analysis, the variables age and education level were selected. Previously available literature noticed that they are important drivers of change [49, 50].

### Ethical consideration

The 2006, 2012 and 2016/17 HDHS survey obtained ethical clearance from the Ethics Committee of ORC Macro Inc. as well as Ethics Boards of the Haitian Bureau of Statistics and the Haitian Ministry of Public Health and Population. During the data collection, either written or verbal consent was provided by the respondents. Since the data was not collected by the authors of this paper, permission was sought from MEASURE DHS website and access to the data was provided after our intent for the request was assessed and approved on May 3, 2022. Data is available on https://dhsprogram.com/data/available-datasets.cfm.

## Results

### Description of sample characteristics

The socio-demographic characteristics of the study population are described in Table 1. About a third of women sampled across the three surveys were 35 years of age or more. More than 40% came from "Ouest" region, and around 15% were from "Artibonite". Although still very high, the proportion of Christian respondents has decreased slightly: it was 92.5% in 2006, 91.5% in 2012, and 89.9% in 2016/17. Conversely, women's educational attainment has improved significantly over time: the proportion of respondents with primary or no formal education decreased from 64.7% in 2006 to 53.2 in 2012 and to 46% in 2016/17, while the proportion of respondents with higher education level more than doubled between 2006 (3.4%) and 2017 (7.6%). Nearly of 55% of women interviewed declared listening to radio almost every day in 2006, compared to 47.9% in 2012, and 39.3% in 2016/17. Approximately 2 out of 10 of them (17.1%) declared watching TV almost every day in 2006, while this proportion was 24.6% in 2012 and 18.1% in 2016/2017. Further, in 2006, 2012 and 2016/17 surveys, about one-third were in the poor (poorest/poorer) wealth index category, and less than 5% were covered by health insurance. In terms of occupation status, majority of women had an income-generating activity across all the survey years.

**Table 1:** Percent distribution of women by selected background characteristics: 2006, 2012, 2016/17

Socio-demographic characteristics	2006		2012		2016/17	
	N	Percentage	N	Percentage	N	Percentage
**Age**						
Less than 25 years	2741	31.8	3760	32.3	3727	31.3
25–34	2877	33.4	4066	34.9	4106	34.5
35 and above	3009	34.9	3811	32.7	4058	34.1
**Place of residence**						
Urban	3983	46.2	5597	48.1	5569	46.8
Rural	4644	53.8	6040	51.9	6322	53.2
**Region**						
Ouest	3713	43.0	5202	44.7	4940	41.5
Sud-Est	373	4.3	466	4.0	605	5.1
Nord	836	9.7	1176	10.1	1274	10.7
Nord-Est	260	3.0	401	3.4	412	3.5
Artibonite	1373	15.9	1685	14.5	1720	14.5
Centre	616	7.1	671	5.8	758	6.4
Sud	513	6.0	784	6.7	790	6.6
Grand'Anse/Nippes	473	5.5	726	6.2	811	6.8
Nord-Ouest	469	5.4	527	4.5	583	4.9
**Religion**						
Christian	7977	92.5	10,642	91.5	10,695	89.9
Non-Christian	650	7.5	995	8.5	1197	10.1
**Education level**						
Primary or less	5585	64.7	6187	53.2	5472	46.0
Secondary	2750	31.9	4744	40.8	5512	46.4
Higher	292	3.4	705	6.1	908	7.6
**Frequency of listening to radio**						
Not at all	792	9.2	1650	14.2	1492	12.5
Less than once a week	1460	16.9	2025	17.4	2861	24.1
At least once a week	1642	19.0	2386	20.5	2871	24.1
Almost every day	4726	54.9	5575	47.9	4668	39.3
**Frequency of watching TV**						
Not at all	3854	44.7	4787	41.2	4614	38.8
Less than once a week	2101	24.4	2421	20.8	3481	29.3
At least once a week	1195	13.9	1561	13.4	1647	13.8
Almost every day	1476	17.1	2864	24.6	2150	18.1
**Wealth index**						
Poorest	1369	15.9	1709	14.7	1801	15.1
Poorer	1386	16.1	1854	15.9	1982	16.7
Middle	1614	18.7	2366	20.3	2324	19.5
Richer	2093	24.3	2708	23.3	2854	24.0
Richest	2164	25.1	3001	25.8	2931	24.6
**Currently working**						
Yes	5155	59.8	6834	58.7	7630	64.2
No	3472	40.2	4802	41.3	4262	35.8
**Marital status**						
Never married	1325	15.4	2629	22.6	3344	28.1
In union	6323	73.3	7806	67.1	7402	62.2
Widowed/Divorced/Separated	980	11.4	1202	10.3	1146	9.6
**Age at first sex**						
Less than 15	1657	19.2	1871	16.1	1820	15.3
15–17	3445	39.9	4924	42.3	5482	46.1
18 and above	3525	40.9	4842	41.6	4590	38.6
**Covered by health insurance**						
Yes	183	2.1	420	3.6	372	3.1
No	8442	97.9	11,210	96.4	11,519	96.9
**Had any STD last 12 months**						
Yes	806	9.4	1367	11.8	927	7.8
No	7801	90.6	10,260	88.2	10,965	92.2
**Number of sexual partners**						
No partner	1257	14.6	1677	14.4	1656	13.9
One partner	7233	83.8	9625	82.8	9832	82.7
Two and above	137	1.6	329	2.8	404	3.4
**Total**	**8627**	**100.0**	**11,637**	**100.0**	**11,892**	**100.0**

Table 1 further shows that majority of respondents were in union, ranging from 73.3% in 2006 to 62.2% in 2016/17. In 2006, most of the women (40.9%) had their first sexual debut in the age group 18 and above, whereas in survey years 2012 and 2016/17, most of them had their first sexual debut in the 15–17 age group (42.3% and 46.1%, respectively). Additionally, 9.4% of women reported getting an STD in 2006, compared to 11.8% in 2012, and 7.8% in 2016/17. Similarly, 1.6%, 2.8%, and 3.4% declared having two or more sexual partners in 2006, 2012, and 2016/17, respectively.

### Trend of HIV testing from 2006 to 2016/17

**Fig. 1:**
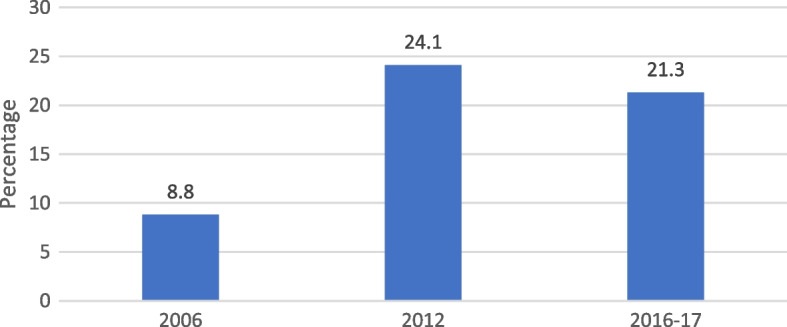
Trends in HIV testing among women interviewed, Haiti DHS 2006, 2012, 2016/17

The Fig. 1 presents the trends of HIV testing among women interviewed across the three surveys. The results revealed that the prevalence of HIV testing increased from 8.8% (95% CI: 8.2 – 9.4) in 2006 to 24.1% (95% CI: 23.3 – 24.9) in 2012. Also, it is observed that this prevalence decreased by 11.6% from 2012 to 2016/17.

### Bivariate associations of HIV testing and socio-demographic characteristics

Bivariate analyses reveal that many individual and community-level variables were consistently associated with HIV testing (Table 2). In all three rounds of the HDHS (2006, 2012, 2016/17), HIV testing was most prevalent among women aged 25 to 34 years, from urban areas and richest households, Christians; with higher education level, who declared listening to radio and watching TV at least once a week or almost every day, who were covered by health insurance, who had an STD, and who reported having two or more sexual partners. Furthermore, HIV testing was much higher among women from "Centre" (11.5%) in 2006, "Nord" (29%) in 2012, and "Nord-Est" (27.4%) in 2016/17. HIV testing was most common among women who never married (9.1%) in 2006, while in 2012 and 2016/17 it was most common among those in union (25.9% and 22%, respectively). Similarly, it was most frequent among respondents who had their first sexual debut in the 15–17 age group (9.6%), whereas it was most prevalent among those who had their first sexual debut in the age group 18 and above in 2012 and 2016/17 (25.2% and 22.2, respectively). Finally, the prevalence of HIV testing was much higher among women who had not an income-generating activity in 2006 (10.1%) and 2012 (25%). However, in 2017, HIV testing was most frequent among women who had an income-generating activity (21.7%).

**Table 2:** Bivariate associations of HIV testing and socio-demographic characteristics

Socio-demographic characteristics	2006		*P*-value	2012		*P*-value	2017		*P*-value
	Yes (N/%)	No (N/%)		Yes (N/%)	No (N/%)		Yes (N/%)	No (N/%)	
**Age**			< 0.001			< 0.001			< 0.001
Less than 25 years	274 (10.0)	2467 (90.0)		972 (25.9)	2788 (74.1)		770 (20.7)	2958 (79.3)	
25–34	346 (12.0)	2532 (88.0)		1240 (30.5)	2826 (69.5)		1093 (26.6)	3014 (73.4)	
35 and above	141 (4.7)	2867 (95.3)		594 (15.6)	3217 (84.4)		672 (16.6)	3387 (83.4)	
**Place of residence**			< 0.001			< 0.001			< 0.001
Urban	478 (12.0)	3505 (88.0)		1627 (29.1)	3969 (70.9)		1432 (25.7)	4138 (74.3)	
Rural	283 (6.1)	4361 (93.9)		1178 (19.5)	4862 (80.5)		1102 (17.4)	5220 (82.6)	
**Region**			0.008			< 0.001			< 0.001
Ouest	352 (9.5)	3361 (90.5)		1365 (26.2)	3837 (73.8)		1121 (22.7)	3818 (77.3)	
Sud-Est	15 (4.0)	358 (96.0)		85 (18.2)	381 (81.8)		88 (14.5)	517 (85.5)	
Nord	71 (8.5)	766 (91.5)		341 (29.0)	834 (71.0)		309 (24.3)	965 (75.7)	
Nord-Est	21 (8.1)	239 (91.9)		93 (23.2)	308 (76.8)		113 (27.4)	299 (72.6)	
Artibonite	112 (8.2)	1261 (91.8)		314 (18.6)	1371 (81.4)		320 (18.6)	1400 (81.4)	
Centre	71 (11.5)	544 (88.5)		129 (19.2)	542 (80.8)		177 (23.4)	581 (76.6)	
Sud	45 (8.8)	469 (91.2)		190 (24.2)	594 (75.8)		135 (17.1)	655 (82.9)	
Grand'Anse/Nippes	35 (7.4)	438 (92.6)		170 (23.4)	556 (76.6)		144 (17.8)	667 (82.2)	
Nord-Ouest	40 (8.5)	429 (91.5)		119 (22.6)	408 (77.4)		126 (21.6)	457 (78.4)	
**Religion**			0.008			0.198			0.053
Christian	722 (9.1)	7254 (90.9)		2582 (24.3)	8060 (75.7)		2304 (21.5)	8390 (78.5)	
Non-Christian	39 (6.0)	612 (94.0)		223 (22.4)	771 (77.6)		229 (19.1)	968 (80.9)	
**Education level**			< 0.001			< 0.001			< 0.001
Primary or less	293 (5.2)	5292 (94.8)		1061 (17.1)	5127 (82.9)		842 (15.4)	4630 (84.6)	
Secondary	393 (14.3)	2357 (85.7)		1426 (30.1)	3318 (69.9)		1375 (24.9)	4137 (75.1)	
Higher	74 (25.4)	217 (74.6)		319 (45.2)	387 (54.8)		317 (34.9)	591 (65.1)	
**Frequency of listening to radio**			< 0.001			< 0.001			< 0.001
Not at all	33 (4.2)	759 (95.8)		264 (16.0)	1386 (84.0)		223 (14.9)	1269 (85.1)	
Less than once a week	111 (7.6)	1349 (92.4)		408 (20.2)	1616 (79.8)		502 (17.5)	2359 (82.5)	
At least once a week	137 (8.3)	1505 (91.7)		580 (24.3)	1807 (75.7)		600 (20.9)	2271 (79.1)	
Almost every day	479 (10.1)	4247 (88.9)		1554 (27.9)	4021 (72.1)		1209 (25.9)	3459 (74.1)	
**Frequency of watching TV**			< 0.001			< 0.001			< 0.001
Not at all	245 (6.4)	3609 (93.6)		873 (18.2)	3914 (81.8)		782 (16.9)	3832 (83.1)	
Less than once a week	177 (8.4)	1924 (91.6)		563 (23.3)	1858 (76.7)		691 (19.9)	2790 (80.1)	
At least once a week	144 (12.1)	1051 (87.9)		466 (29.9)	1095 (70.1)		471 (28.6)	1175 (71.4)	
Almost every day	194 (13.1)	1282 (86.9)		903 (31.5)	1960 (68.5)		590 (27.4)	1560 (72.6)	
**Wealth index**			< 0.001			< 0.001			< 0.001
Poorest	49 (3.6)	1319 (96.4)		233 (13.6)	1475 (86.4)		215 (11.9)	1586 (88.1)	
Poorer	92 (6.6)	1295 (93.4)		318 (17.2)	1536 (82.8)		290 (14.6)	1691 (85.4)	
Middle	109 (6.8)	1505 (93.2)		571 (24.1)	1795 (75.9)		523 (22.5)	1802 (77.5)	
Richer	190 (9.1)	1903 (90.9)		731 (27.0)	1977 (73.0)		671 (23.5)	2183 (76.5)	
Richest	320 (14.8)	1843 (85.2)		952 (31.7)	2048 (68.3)		835 (28.5)	2096 (71.5)	
**Currently working**			< 0.001			0.048			0.213
Yes	409 (7.9)	4747 (92.1)		1603 (23.5)	5231 (76.5)		1652 (21.7)	5978 (78.3)	
No	352 (10.1)	3119 (89.9)		1203 (25.0)	3600 (75.0)		881 (20.7)	3380 (79.3)	
**Marital status**			0.282			< 0.001			0.061
Never married	120 (9.1)	1204 (90.9)		560 (21.3)	2068 (78.7)		682 (20.4)	2662 (79.6)	
In union	567 (9.0)	5756 (91.0)		2020 (25.9)	5786 (74.1)		1627 (22.0)	5774 (78.0)	
Widowed/Divorced/Separated	73 (7.5)	906 (92.5)		225 (18.7)	977 (81.3)		225 (19.6)	921 (80.4)	
**Age at first sex**			0.098			< 0.001			0.014
Less than 15	133 (8.0)	1525 (92.0)		384 (20.5)	1487 (79.5)		344 (18.9)	1476 (81.1)	
15–17	331 (9.6)	3114 (90.4)		1200 (24.4)	3724 (75.6)		1170 (21.3)	4312 (78.7)	
18 and above	297 (8.4)	3227 (91.6)		1222 (25.2)	3620 (74.8)		1020 (22.2)	3570 (77.8)	
**Covered by health insurance**			< 0.001			< 0.001			< 0.001
Yes	46 (25.1)	137 (74.9)		148 (35.2)	272 (64.8)		145 (38.9)	228 (61.1)	
No	715 (8.5)	7728 (91.5)		2658 (23.7)	8552 (76.3)		2389 (20.7)	9130 (79.3)	
**Had any STD last 12 months**			< 0.001			< 0.001			< 0.001
Yes	119 (14.8)	687 (85.2)		412 (30.1)	955 (69.9)		364 (39.3)	563 (60.7)	
No	640 (8.2)	7163 (91.8)		2392 (23.3)	7868 (76.7)		2170 (19.8)	8794 (80.2)	
**Number of sexual partners**			< 0.001			< 0.001			< 0.001
No partner	76 (6.0)	1181 (94.0)		290 (17.3)	1387 (82.7)		248 (15.0)	1408 (85.0)	
One partner	660 (9.1)	6572 (90.9)		2428 (25.2)	7197 (74.8)		2184 (22.2)	7648 (77.8)	
Two and above	24 (17.5)	113 (82.5)		86 (26.2)	242 (73.8)		102 (25.2)	302 (74.8)	
**Total**	**761 (8.8)**	**7866 (91.2)**		**2806 (24.1)**	**8831 (75.9)**		**2534 (21.3)**	**9358 (78.7)**	

### Decomposition results

Change observed in HIV testing prevalence between 2006 and 2016/17 is driven by combination of the two effects. Basic decomposition showed that compositional factors explain 17.5% of the change, while behavioral factors account for 82.5% (Appendix Table A1). The proportion of women with no formal education declined (from 64.7% to 46%) while there was a rise in the proportions of women with secondary (31.9 to 46.4%) and higher level (3.4 to 7.6%) between 2006 and 2016/17 (Table 1). In addition, we found that HIV testing prevalence increased during this period, regardless of education level and age group (Table 2). Advanced decomposition demonstrated that the variation of HIV testing prevalence is primarily due to the increase in the proportion of women aged 35 years and above (8.1%) and to an improvement in the educational level of women in the 35 + age group (9.4%) (Appendix Table A1). The women aged 35 and above with good education levels constituted the dominant social group driving change.

### Individual and community-level factors associated with HIV testing (The fixed effect analysis)

Tables 3, 4 and 5 display results from the multilevel logistic regressions. In 2006, it is revealed that women aged less than 25 years (aOR = 1.78; 95% CI: 1.38 – 2.31) and 25–34 years old (aOR = 2.12; 95% CI: 1.70 – 2.65) had higher odds of HIV testing compared to the 35 years and above. Respondents with secondary (aOR = 2.21; 95% CI: 1.79 – 2.72) and higher (aOR = 4.57; 95% CI: 3.14 – 6.63) education levels were at least 2 times more likely to be tested for HIV than those with primary or no formal education. Being in the "richest" category of household wealth index was associated with increased odds (aOR = 1.57; 95% CI: 1.24 – 2.00) of HIV testing compared to being in the "richer" category. Furthermore, the likelihood of HIV testing among women who had never married (aOR = 0.59; 95% CI: 0.46 – 0.76) was decreased by 41% than those who were in union. Having her first sexual debut in the 15–17 age group was associated with 21% higher odds (aOR = 1.21; 95% CI: 1.01 – 1.46) of HIV testing, whereas having no sexual partner was associated with 36% lower odds (aOR = 0.64; 95% CI: 0.47 –0.87) of HIV testing. Similarly, being covered by health insurance (aOR = 1.74; 95% CI: 1.16 – 2.61) and getting an STD in last 12 months (aOR = 1.75; 95% CI: 1.39 – 2.22) increased the odds of HIV testing by 1.7 times. Women from urban areas (aOR = 1.56; 95% CI: 1.12 – 2.15) were found to have a higher probability of HIV testing. Likewise, respondents from "Artibonite" (aOR = 1.55; 95% CI: 1.02 – 2.37), "Centre" (aOR = 3.14; 95% CI: 1.92 – 5.14), "Sud" (aOR = 1.77; 95% CI: 1.07 – 2.95), "Grand’Anse/Nippes" (aOR = 1.79; 95% CI: 1.07 – 2.98), and "Nord-Ouest" (aOR = 1.70; 95% CI: 1.02 – 2.85) were more likely to be tested for HIV than their counterparts from "Ouest" region.

**Table 3:** Multilevel regression for HIV testing by selected socio-demographic characteristics (HDHS 2006)

Socio-demographic characteristics	Model-0 ICC = 18.39%	Model-I aOR (95% CI)	Model-II aOR (95% CI)	Model-III aOR (95% CI)
**Age**				
Less than 25 years		1.80 (1.39—2.32)***		1.78 (1.38—2.31)***
25–34		2.13 (1.71—2.67)***		2.12 (1.70—2.65)***
Ref = 35 and above				
**Religion**				
Non-Christian		0.87 (0.61—1.25)		0.89 (0.62—1.28)
Ref = Christian				
**Education level**				
Secondary		2.22 (1.81—2.74)***		2.21 (1.79—2.72)***
Higher		4.64 (3.19—6.74)***		4.57 (3.14—6.63)***
Ref = Primary or less				
**Frequency of listening to radio**				
Not at all		0.86 (0.57—1.30)		0.84 (0.56—1.27)
Less than once a week		1.17 (0.90—1.51)		1.15 (0.89—1.49)
At least once a week		1.01 (0.80—1.25)		1.01 (0.81—1.27)
Ref = Almost every day				
**Frequency of watching TV**				
Less than once a week		0.85 (0.66—1.08)		0.87 (0.68—1.11)
At least once a week		0.94 (0.70—1.27)		0.98 (0.73—1.33)
Almost every day		0.84 (0.62—1.14)		0.88 (0.64—1.20)
Ref = Not at all				
**Wealth index**				
Poorest		0.48 (0.32—0.74)**		0.50 (0.31—0.80)**
Poorer		1.03 (0.74—1.44)		1.10 (0.75—1.60)
Middle		0.97 (0.73—1.30)		0.98 (0.72—1.33)
Richest		1.57 (1.23—1.99)***		1.57 (1.24—2.00)***
Ref = Richer				
**Currently working**				
No		1.13 (0.95—1.35)		1.14 (0.96 – 1.36)
Ref = Yes				
**Marital status**				
Never married		0.59 (0.45—0.76)***		0.59 (0.46—0.76)***
Widowed/Divorced/Separated		1.21 (0.88—1.65)		1.20 (0.88—1.64)
Ref = In union				
**Age at first sex**				
Less than 15		1.13 (0.89—1.45)		1.13 (0.89—1.45)
15–17		1.22 (1.01—1.47)*		1.21 (1.01—1.46)*
Ref = 18 and above				
**Covered by health insurance**				
Yes		1.74 (1.16—2.61)**		1.74 (1.16—2.61)**
Ref = No				
**Had any STD last 12 months**				
Yes		1.76 (1.39—2.23)***		1.75 (1.39—2.22)***
No				
**Number of sexual partners**				
No partner		0.64 (0.47—0.87)**		0.64 (0.47—0.87)**
Two and above		1.56 (0.94—2.59)		1.57 (0.95—2.59)
Ref = One partner				
**Place of residence**				
Urban			2.77 (2.10—3.65)***	1.56 (1.12—2.15)**
Ref = Rural				
**Region**				
Sud-Est			0.63 (0.32—1.24)	0.80 (0.40—1.57)
Nord			1.12 (0.71—1.76)	1.44 (0.92—2.27)
Nord-Est			1.11 (0.61—2.01)	1.65 (0.90—3.03)
Artibonite			1.13 (0.74—1.73)	1.55 (1.02—2.37)*
Centre			2.00 (1.24—3.24)**	3.14 (1.92—5.14)***
Sud			1.49 (0.89—2.48)	1.77 (1.07—2.95)*
Grand'Anse/Nippes			1.20 (0.73—1.96)	1.79 (1.07—2.98)*
Nord-Ouest			1.32 (0.79—2.21)	1.70 (1.02—2.85)*
Ref = Ouest				

^*^p < .05. **p < .01. ***p < .001

**Table 4:** Multilevel regression for HIV testing by selected socio-demographic characteristics (HDHS 2012)

Socio-demographic characteristics	Model-0 ICC = 8.45%	Model-I aOR (95% CI)	Model-II aOR (95% CI)	Model-III aOR (95% CI)
**Age**				
Less than 25 years		1.07 (0.95—1.20)		1.07 (0.95—1.20)
35 and above		0.47 (0.41—0.53)***		0.47 (0.41—0.53)***
Ref = 25–34				
**Religion**				
Non-Christian		0.96 (0.81—1.14)		1.01 (0.84 (1.19)
Ref = Christian				
**Education level**				
Secondary		1.63 (1.45—1.82)***		1.62 (1.44—1.81)***
Higher		3.19 (2.60—3.90)***		3.16 (2.58—3.87)***
Ref = Primary or less				
**Frequency of listening to radio**				
Not at all		0.79 (0.67—0.93)**		0.79 (0.67—0.93)**
Less than once a week		0.92 (0.80—1.06)		0.92 (0.80—1.06)
At least once a week		0.95 (0.84—1.08)		0.95 (0.85—1.08)
Ref = Almost every day				
**Frequency of watching TV**				
Less than once a week		1.04 (0.90—1.20)		1.06 (0.92—1.22)
At least once a week		1.27 (1.08—1.49)**		1.30 (1.11—1.54)**
Almost every day		1.11 (0.95—1.30)		1.15 (0.98—1.35)
Ref = Not at all				
**Wealth index**				
Poorest		0.65 (0.53—0.79)***		0.66 (0.53—0.81)***
Poorer		0.77 (0.65—0.92)**		0.79 (0.66—0.95)*
Richer		1.07 (0.92—1.24)		1.03 (0.88—1.20)
Richest		1.20 (1.01—1.43)*		1.15 (0.96—1.37)
Ref = Middle				
**Currently working**				
No		1.04 (0.94—1.14)		1.03 (0.94—1.14)
Ref = Yes				
**Marital status**				
Never married		0.44 (0.38—0.50)***		0.43 (0.38—0.50)***
Widowed/Divorced/Separated		0.78 (0.65—0.94)**		0.78 (0.65—0.94)**
Ref = In union				
**Age at first sex**				
Less than 15		0.88 (0.77—1.02)		0.88 (0.76—1.01)
18 and above		1.02 (0.92—1.14)		1.03 (0.93—1.14)
Ref = 15–17				
**Covered by health insurance**				
Yes		1.01 (0.80—1.27)		1.02 (0.81—1.28)
Ref = No				
**Had any STD last 12 months**				
Yes		1.32 (1.15—1.51)***		1.32 (1.15—1.51)***
No				
**Number of sexual partners**				
No partner		0.80 (0.68—0.94)**		0.80 (0.68—0.93)**
Two and above		0.84 (0.65—1.10)		0.84 (0.64—1.10)
Ref = One partner				
**Place of residence**				
Urban			1.80 (1.55—2.08)***	1.20 (1.01—1.43)*
Ref = Rural				
**Region**				
Sud-Est			0.82 (0.59—1.13)	1.05 (0.76—1.44)
Nord			1.37 (1.08—1.72)**	1.56 (1.25—1.96)***
Nord-Est			0.99 (0.72—1.36)	1.22 (0.89—1.66)
Artibonite			0.76 (0.60—0.95)*	0.87 (0.69—1.09)
Centre			0.85 (0.64—1.14)	1.15 (0.86—1.54)
Sud			1.19 (0.91—1.56)	1.37 (1.05—1.78)*
Grand'Anse/Nippes			1.15 (0.89—1.47)	1.57 (1.22—2.03)***
Nord-Ouest			1.03 (0.77—1.38)	1.30 (0.97—1.75)
Ref = Ouest				

^*^p < .05. **p < .01. ***p < .001

**Table 5:** Multilevel regression for HIV testing by selected socio-demographic characteristics (HDHS 2016/17)

Socio-demographic characteristics	Model-0 ICC = 7.53%	Model-I aOR (95% CI)	Model-II aOR (95% CI)	Model-III aOR (95% CI)
**Age**				
Less than 25 years		1.45 (1.24—1.69)***		1.44 (1.23—1.68)***
25–34		1.59 (1.42—1.80)***		1.59 (1.41—1.79)***
Ref = 35 and above				
**Religion**				
Non-Christian		0.99 (0.84—1.18)		1.02 (0.87—1.22)
Ref = Christian				
**Education level**				
Secondary		1.45 (1.24—1.69)***		1.45 (1.29—1.64)***
Higher		2.05 (1.68—2.51)***		2.08 (1.70—2.54)***
Ref = Primary or less				
**Frequency of listening to radio**				
Not at all		0.76 (0.64—0.91)**		0.76 (0.64—0.91)**
Less than once a week		0.82 (0.72—0.93)**		0.82 (0.72—0.93)**
At least once a week		0.87 (0.77—0.99)*		0.88 (0.78—0.99)*
Ref = Almost every day				
**Frequency of watching TV**				
Less than once a week		0.96 (0.84—1.09)		0.97 (0.85—1.11)
At least once a week		1.25 (1.06—1.48)**		1.27 (1.08—1.50)**
Almost every day		1.08 (0.91—1.27)		1.09 (0.92—1.30)
Ref = Not at all				
**Wealth index**				
Poorest		0.61 (0.50—0.76)***		0.62 (0.50—0.78)***
Poorer		0.72 (0.60—0.87)***		0.74 (0.61—0.91)**
Midlle		1.04 (0.90—1.21)		1.07 (0.92—1.24)
Richest		1.12 (0.98—1.29)		1.13 (0.98—1.30)
Ref = Richer				
**Currently working**				
No		0.94 (0.84—1.05)		0.93 (0.83—1.04)
Ref = Yes				
**Marital status**				
Never married		0.66 (0.58—0.75)***		0.66 (0.58—0.76)***
Widowed/Divorced/Separated		1.05 (0.87—1.25)		1.05 (0.88—1.26)
Ref = In union				
**Age at first sex**				
Less than 15		0.94 (0.81—1.08)		0.93 (0.81—1.07)
18 and above		1.01 (0.91—1.13)		1.01 (0.91—1.12)
Ref = 15–17				
**Covered by health insurance**				
Yes		1.44 (1.13—1.82)**		1.44 (1.14 -1.83)**
Ref = No				
**Had any STD last 12 months**				
Yes		2.60 (2.24—3.03)***		2.59 (2.23—3.01)***
No				
**Number of sexual partners**				
No partner		0.69 (0.59—0.81)***		0.69 (0.58—0.81)***
Two and above		0.97 (0.76—1.24)		0.98 (0.76—1.26)
Ref = One partner				
**Place of residence**				
Urban			1.68 (1.46—1.94)***	1.13 (0.96—1.32)
Ref = Rural				
**Region**				
Sud-Est			0.76 (0.55—1.03)	0.83 (0.61—1.13)
Nord			1.27 (1.01—1.60)*	1.46 (1.17—1.81)**
Nord-Est			1.46 (1.08—1.97)**	1.77 (1.32—2.37)***
Artibonite			0.89 (0.72—1.10)	1.10 (0.90—1.36)
Centre			1.30 (1.01—1.69)*	1.62 (1.25—2.08)***
Sud			0.89 (0.68—1.17)	1.04 (0.80—1.36)
Grand'Anse/Nippes			0.93 (0.72—1.19)	1.13 (0.88—1.45)
Nord-Ouest			1.18 (0.90—1.56)	1.43 (1.09—1.87)**
Ref = Ouest				

^*^p < .05. **p < .01. ***p < .001

In multivariate analysis in 2012, women with secondary (aOR = 1.62; 95% CI: 1.44 – 1.81) and higher (aOR = 3.16; 95% CI: 2.58 – 3.87) education levels; and who had an STD (aOR = 1.32; 95% CI: 1.15 – 1.51) were associated with greater odds of HIV testing. The results also indicated that women aged 35 and above (aOR = 0.47; 95% CI: 0.41 – 0.53), from poorest (aOR = 0.66; 95% CI: 0.53 – 0.81) and poorer (aOR = 0.79; 95% CI: 0.66 – 0.95) households, who had been never married (aOR = 0.43; 95% CI: 0.38 – 0.50) or widowed/divorced/separated (aOR = 0.78; 95% CI: 0.65 – 0.94), and with no sexual partner (aOR = 0.80; 95% CI: 0.68 – 0.93) were less likely to be tested for HIV. Women from urban areas (aOR = 1.20; 95% CI: 1.01 – 1.43) were found to have a higher likelihood of HIV testing. Compared to participants from "Ouest", those who came from "Nord", "Sud", and "Grand’Anse/Nippes" had 1.6 (aOR = 1.56; 95% CI: 1.25 – 1.96), 1.4 (aOR = 1.37; 95% CI: 1.05 – 1.78), and 1.6 (aOR = 1.57; 95% CI: 1.22 – 2.03) greater odds of HIV testing, respectively. Besides, being exposed to TV at least once a week was associated with 30% higher odds (aOR = 1.30; 95% CI: 1.11 – 1.54) of HIV testing, while not being exposed to radio at all was associated with 21% lower odds (aOR = 0.79; 95% CI: 0.67 –0.93) of HIV testing.

As for 2016/17, the same trends are observed. Compared to women aged less than 25 years (aOR = 1.44; 95% CI: 1.23 – 1.68) and 25–34 years old (aOR = 1.59; 95% CI: 1.41 – 1.79), those aged 35 and above were less likely to tested for HIV. Respondents with secondary (aOR = 1.45; 95% CI: 1.29 – 1.64) and higher (aOR = 2.08; 95% CI: 1.70 – 2.54) education levels were more likely to be tested for HIV as compared to primary or uneducated women. Women in "poorest" (aOR = 0.62; 95% CI: 0.50 – 0.78) and "poorer" (aOR = 0.74; 95% CI: 0.61 – 0.91) categories of household wealth index, who had been never married (aOR = 0.66; 95% CI: 0.58 – 0.76), with no sexual partner (aOR = 0.69; 95% CI: 0.58 – 0.81) had a lower likelihood of HIV testing as compared to their counterparts in "richer" category of household wealth index, in union, and with one sexual partner, respectively. Being covered by health insurance (aOR = 1.44; 95% CI: 1.14 – 1.83) and getting an STD (aOR = 2.59; 95% CI: 2.23 – 3.01) were associated with increased odds of HIV testing. Region-wise, women from "Nord" (aOR = 1.46; 95% CI: 1.17 – 1.81), "Nord-Est" (aOR = 1.77; 95% CI: 1.32 – 2.37), "Centre" (aOR = 1.62; 95% CI: 1.25 – 2.08) and "Nord-Ouest" (aOR = 1.43; 95% CI: 1.09 – 1.87) had greater odds of HIV testing as compared to those from "Ouest". Exposure to mass media also had a positive relationship with HIV testing, indicating that participants who reported listening to radio less than once a week (aOR = 0.82; 95% CI: 0.72 – 0.93) or at least once a week (aOR = 0.88; 95% CI: 0.78 – 0.99) were 12% to 18% less likely to be tested for HIV than those who declared listening to radio almost every day. Similarly, women who reported watching TV at least once a week were 1.3 times (aOR = 1.27; 95% CI: 1.08 – 1.50) more likely to be tested as compared to those who had not listened to the radio at all.

### Measures of variation (Random effect analysis)

The results show that the variance for the null model (Model-0) was 0.74 (95% CI: 0.53 – 1.03) in 2006, 0.30 (95% CI: 0.23 – 0.39) in 2012, and 0.27 (95% CI: 0.20 – 0.35) in 2016/17, respectively (Table 6, Appendix Tables A2-A3). The null model also reveals that 18.4% (in 2006), 8.5% (in 2012), and 7.5% (in 2016/17) of the total variance in HIV testing was attributed to between-cluster variation. Besides, the PCV in the final model (Model-III) indicates that 33.8%, 43.3%, and 48.2% of the variability in HIV testing was explained by both individual and community-level characteristics, respectively in 2006, 2012, and 2016/17.

**Table 6:** Measure of variation for HIV testing in Haiti, HDHS 2006

Measure of variation	Model-0	Model-I	Model-II	Model-III
Variance	0.74 (0.53—1.03)	0.59 (0.42—0.84)	0.57 (0.40—0.81)	0.49 (0.34—0.72)
ICC (%)	18.39	15.30	14.82	13.06
PCV (%)	Reference	20.27	22.97	33.78
Model fitness				
Log-likelihood	-2459.97	-2280.88	-2428.71	-2265.05
AIC	4923.94	4613.77	4879.43	4600.11

*ICC* Intra-class Correlation Coefficient

*PCV* Proportional Change in Variance

*AIC* Akaike Information Criterion

## Discussion

Using the last three HDHS (2006, 2012, and 2016/17), this paper examines the trend and explores factors associated with HIV testing among women in Haiti. Over the years, a growing trend has been observed in the use of HIV testing: it increased more than twofold from 2006 (8.8%) to 2017 (21.3%). Demographic decomposition analysis shows that this increase is greatly driven by the improvement in the level of education of women during this period, especially those aged 35 and above who form the dominant social group driving change. The efforts of the Haitian Ministry of Public Health and Population and its partners such as UNFPA, WHO, UNICEF, and USAID, over the past two decades, through programs aimed at improving access to sexual and reproductive health services for all women, have also played a significant role [51, 52]. However, it should be noted that between 2012 and 2016/17, there was a slight decline in the prevalence of HIV testing, while the latest HDHS report shows an upward trend among women of reproductive age during this period. Disparities in findings may be partly due to a sampling effect, as our study was restricted to women who had already had sex at the time of the surveys. Additionally, results from this study both support and contradict findings from other researchers on HIV testing among women.

Similar to prior studies [39, 53], our data demonstrate that region is an independent predictor for HIV testing. Women from "Nord", "Nord-Est", "Centre", "Sud", "Grand’Anse/Nippes", and "Nord-Ouest" were found to be more likely to use HIV testing than their counterparts from "Ouest". A possible explanation for this observation is that the prevalence of HIV is generally higher in most of these regions than in the "Ouest". To prevent the spread of infection, many awareness campaigns have been implemented specifically in these areas, promoting HIV testing and providing free antiretroviral (ARV) treatment and sexual health services [54–56].

Place of residence is a strong predictor of HIV testing. Our results highlight that women from urban communities had increased odds of being tested, which is consistent with previous studies in Malawi [20] and Ethiopia [16]. This reflects different factors. First, women living in rural areas in Haiti have less access to sexual health information and services than urban women [31]. Second, rural areas are highly marginalized and the community has poor access to education. Third, in some remote rural areas, women need to travel long distances to reach health facilities [57, 58], which constitutes a real barrier for them. Lastly, stigma surrounding STIs may make rural women less likely to seek testing [59].

A high education level has been found to increase the likelihood of HIV testing. This finding is supported by various other studies [10, 60, 61]. Educated women may be more exposed to STIs transmission and prevention information and have a better understanding of the benefits of testing [40]. Besides, they can influence their partner's sexual behavior and encourage them to get tested [25].

Women in union are more likely to test for HIV than those who have never married. This has been confirmed by studies conducted in Gambia [40], the Northwest of Ethiopia [62], and Thailand [41]. This may be explained by the perceived risk associated with being infected in previous or current relationships, as well as the compulsory testing carried out for partners who intend to marry [41]. Another plausible reason could be the fact that women in union are more likely to visit antenatal health services. In Haiti, antenatal care in public health facility includes free HIV testing for women [63].

The results further reveal that women aged 35 and above have lower odds of being tested for HIV compared to their counterparts. This finding is at variance with a study conducted in Lesotho [22]. The fact that younger women are increasingly the target of sexual education and HIV testing programs in Haiti could greatly explain this association [38].

One of the interesting findings of our study is that women who reported watching TV/listening to radio almost every day or at least once a week are more likely to test for HIV compared to their counterparts who reported no exposure to these mass media, which corroborates previous findings [64]. Mass media is the major source of information, and most powerful for addressing a large group of people to change community awareness, attitude and practice towards HIV [65]. Exposure to mass media can improve the sexual health knowledge of Haitian women through delivering a repeated message about how STIs are transmitted and how to prevent them. The importance of mass media in health promotion and disease prevention is well-documented, as both routine exposure to and strategic use of mass media play a significant role in promoting awareness, increasing knowledge, and changing health behaviors [66]. However, note that the variables that measured mass media exposure (frequency of listening to radio, frequency of watching TV) were not statistically significant in 2006.

Being in a higher economic quintile is associated with an increased likelihood of HIV testing. Other studies have also found a positive association between wealth index and HIV testing [11, 64, 67], possibly because women in higher economic quintile have a better education level, coupled with the economic privilege to access HIV testing services than those in the lowest quintiles [40].

Furthermore, women who had any STD last 12 months were identified to be positively associated with HIV testing. According to previous studies [41, 68], this finding may be attributed to doctors asking these women who are sexually active to be tested regularly to protect themselves and their partners, which increases the chances of HIV testing [40, 41].

Women were covered by health insurance had higher odds of HIV testing as compared to those who were not covered. This result is in line with former studies [40, 69]. Health insurance plays an important role in ensuring health equity. Being covered by health insurance may increase health care service utilization, which could raise women’s awareness towards HIV testing [69].

Respondents who had a sexual partner were more likely to undergo HIV testing than those without a partner. This is confirmed by studies conducted in China [70], and Nepal [64]. This might be justified by the fact that women with no sexual partner do not perceive themselves at risk and therefore diminish the importance of HIV testing, whereas those with one or more sexual partners are more exposed to HIV infection, which motivates them to seek testing services regularly [25].

Finally, women who initiated sexual activity at age 18 or older had a lower chance of being tested for HIV compared to their counterparts. This is validated by studies conducted elsewhere [16, 20, 25]. Young age at first sexual intercourse is correlated with a higher risk of contracting various STDs and engaging in risky sexual practices that could lead to HIV infection [25]. Consequently, women who had their first sexual intercourse before age 18 may have more frequent fear, which prompts them to know their HIV status [25, 71].

Strengths and limitations of the study.

This study has several strengths. It contributes to scholarship on trends and factors associated with HIV testing among women in Haiti by analyzing data from a nationally representative survey with a large sample, high precision, and generalizability. Nonetheless, our findings might be limited by four factors. First, as this study had a cross-sectional design, a cause-and-effect relationship cannot be established. Second, the HIV testing data are self-reported and thus subject to recall and social desirability bias. Third, this study only focused on women aged of 15–49 years who had made their sexual debut, excluded women who have not yet had sex and men. Lastly, the analysis was limited to the available variables included in the 2006, 2012, and 2016/17 HDHS.

## Conclusion

Based on the results of our study, the prevalence of HIV testing remains low among women in Haiti, despite the implementation of numerous sexual health programs by the Ministry of Public Health and Population and its partners (WHO, UNFPA, USAID, UNICEF…) over the past two decades. Further efforts are required by the Haitian government to meet the first target set by UNAIDS, which aims to ensure that 95% of people know their HIV serostatus by 2030. According to the World Health Organization (WHO), offering a variety of HIV testing approaches is a crucial strategy for achieving testing, prevention, and treatment goals, ultimately contributing to maintaining low HIV incidence, stated Dr. Meg Doherty, Director of WHO Global HIV, Hepatitis, and STI Programs: "Countries are urged to expand the utilization of HIV self-testing and implement social network testing approaches to address gaps in coverage related to access, stigma, awareness, or affordability". In alignment with WHO recommendations, the Haitian government must prioritize health education targeting vulnerable groups, such as youth, women in union, and those with no formal education, to achieve this goal. Furthermore, they must conduct mobile HIV testing campaigns, providing HIV testing services to communities via mobile clinics, reducing geographic barriers to accessing HIV testing services. This approach enables early detection and treatment. It's also worth mentioning that women of childbearing age are not a homogeneous group, and that each region has its own specific characteristics. Consequently, Haitian government programs must take these specificities into account so that all women and girls can fully enjoy their sexual and reproductive rights. In Haiti, the government usually uses mass media campaigns for sexual health promotion. However, to reach women from rural areas and poor households with limited access to electricity, the government should mobilize community-based communication strategies, including local existing social networks and interpersonal communication.

### Supplementary Information

**Additional file 1:** **Table A1.** Results of the basic and advanced decompositions. **Table A2.** Measure of variation for HIV testing in Haiti, HDHS 2012. **Table A3.** Measure of variation for HIV testing in Haiti, HDHS 2016/17.

## Data Availability

The data used in this study is publicly available at: https://dhsprogram.com/data/availabledatasets.cfm.
