# Supplementary material for: Trends and factors associated with recent HIV testing among women in Haiti: a cross-sectional study using data from nationally representative surveys
Source: BMC Infect Dis. 2024 Jan 11;24:74. doi: 10.1186/s12879-023-08936-z (PMC10782569; doi:10.1186/s12879-023-08936-z)
Supplement: Supplementary file 1 — Additional file 1: Table A1. Results of the basic and advanced decompositions. Table A2. Measure of variation for HIV testing in Haiti, HDHS 2012. Table A3. Measure of variation for HIV testing in Haiti, HDHS 2016/17. [file 12879_2023_8936_MOESM1_ESM.docx]

**Appendix**

Table A1.- Results of the basic and advanced decompositions

| **Education level** | | Primary or less | Secondary | Higher | Total | **Total contribution** |
| --- | --- | --- | --- | --- | --- | --- |
| **HDHS 2006** | % of total population | 0.65 | 0.32 | 0.03 | 1.00 | **-** |
|  | HIV testing prevalence | 5.20 | 14.30 | 25.40 | 8.79 | **-** |
| **HDHS 2016/17** | % of total population | 0.46 | 0.46 | 0.08 | 1.00 | **-** |
|  | HIV testing prevalence | 15.40 | 24.90 | 34.90 | 21.29 | **-** |
| **Basic decomposition** | Increase due to change in composition | -1.93 | 2.84 | 1.27 | 2.18 | **17.50%** |
|  | Increase due to change in behavior | 5.65 | 4.15 | 0.52 | 10.32 | **82.50%** |
|  | Contribution by group | 3.72 | 6.99 | 1.79 | 12.50 | **-** |
|  | Relative contribution (%) | 29.80 | 55.90 | 14.30 | 100.00 | **-** |
| **Advanced decomposition of behavioral effects** | Baseline effect | 5.98 | 4.23 | 0.59 | 10.81 | **86.35%** |
|  | Differentiating effect β | -0.19 | -0.27 | -0.06 | -0.53 | **-4.20%** |
|  | Residual effect | -0.14 | 0.20 | -0.01 | 0.04 | **0.35%** |
|  | Contribution by group | 5.65 | 4.15 | 0.52 | 10.32 |  |
|  | Relative contribution (%) | 54.75 | 40.21 | 5.04 | 100.00 |  |
| **Advanced decomposition of compositional effects** | Effect of changing distribution of women aged 35 and above^a^ | -1.89 | 1.63 | 1.26 | 1.00 | **8.10%** |
|  | Effect of changing distribution of educational attainment for women aged 35 and above | -0.0 | 1.21 | 0.00 | 1.18 | **9.40%** |
|  | Contribution by group | -1.93 | 2.84 | 1.27 | 2.18 | **-** |
|  | Relative contribution (%) | -88.53 | 130.28 | 58.26 | 100.00 | **-** |

^a^In the advanced decomposition analysis, the variable age was divided into "less than 35" and "35 and above"

| Table A2.- Measure of variation for HIV testing in Haiti, HDHS 2012 | | | | |
| --- | --- | --- | --- | --- |
| Variance | 0.30 (0.23 - 0.39) | 0.20 (0.15 - 0.27) | 0.21 (0.16 - 0.28) | 0.17 (0.12 - 0.24) |
| ICC (%) | 8.45 | 5.75 | 5.95 | 4.90 |
| PCV (%) | Reference | 33.33 | 30.00 | 43.33 |
| Model fitness |  |  |  |  |
| Log-likelihood | -6300.76 | -5922.42 | -6253.35 | -5904.77 |
| AIC | 12605.52 | 11896.84 | 12528.70 | 11879.54 |
| Table A3.- Measure of variation for HIV testing in Haiti, HDHS 2016/17 | | | | |
| Variance | 0.27 (0.20 - 0.35) | 0.17 (0.12 - 0.24) | 0.19 (0.14 - 0.26) | 0.14 (0.09 - 0.20) |
| ICC (%) | 7.53 | 4.88 | 5.46 | 4.02 |
| PCV (%) | Reference | 37.04 | 29.63 | 48.15 |
| Model fitness |  |  |  |  |
| Log-likelihood | -6061.24 | -5760.10 | -6019.20 | -5739.87 |
| AIC | 12126.49 | 11572.20 | 12060.41 | 11549.74 |
